# A Review on Biological Effects of Ultrasounds: Key Messages for Clinicians

**DOI:** 10.3390/diagnostics13050855

**Published:** 2023-02-23

**Authors:** Carla Maria Irene Quarato, Donato Lacedonia, Michela Salvemini, Giulia Tuccari, Grazia Mastrodonato, Rosanna Villani, Lucia Angela Fiore, Giulia Scioscia, Antonio Mirijello, Annarita Saponara, Marco Sperandeo

**Affiliations:** 1 Department of Medical and Surgical Sciences, Institute of Respiratory Diseases, Policlinico Universitario “Riuniti” di Foggia, University of Foggia, 71122 Foggia, Italy; 2 Department of Medical and Surgical Sciences, Institute of Geriatric, Policlinico Universitario “Riuniti” di Foggia, University of Foggia, 71122 Foggia, Italy; 3 Department of Basic Medical Science, Neuroscience and Sensory Organs, Institute of Sports Medicine, University “Aldo Moro” of Bari, 70122 Bari, Italy; 4 Department of Medical and Surgical Sciences, Institute of Internal Medicine, Liver Unit, Policlinico Universitario “Riuniti” di Foggia, University of Foggia, 71122 Foggia, Italy; 5 Department of Internal of Medicine, IRCCS Fondazione Casa Sollievo della Sofferenza, 71013 San Giovanni Rotondo, Italy; 6 Unità Sanitaria Locale (ASL) di Potenza, 85100 Potenza, Italy; 7 Unit of Interventional and Diagnostic Ultrasound of Internal Medicine, IRCCS Fondazione Casa Sollievo della Sofferenza, 71013 San Giovanni Rotondo, Italy

**Keywords:** ultrasound, diagnostic imaging, heating, cavitation, radiation force, biological effects, biological hazard, safety, medical practice

## Abstract

Ultrasound (US) is acoustic energy that interacts with human tissues, thus, producing bioeffects that may be hazardous, especially in sensitive organs (i.e., brain, eye, heart, lung, and digestive tract) and embryos/fetuses. Two basic mechanisms of US interaction with biological systems have been identified: thermal and non-thermal. As a result, thermal and mechanical indexes have been developed to provide a means of assessing the potential for biological effects from exposure to diagnostic US. The main aims of this paper were to describe the models and assumptions used to estimate the “safety” of acoustic outputs and indices and to summarize the current state of knowledge about US-induced effects on living systems deriving from in vitro models and in vivo experiments on animals. This review work has made it possible to highlight the limits associated with the use of the estimated safety values of thermal and mechanical indices relating above all to the use of new US technologies, such as contrast-enhanced ultrasound (CEUS) and acoustic radiation force impulse (ARFI) shear wave elastography (SWE). US for diagnostic and research purposes has been officially declared safe, and no harmful biological effects in humans have yet been demonstrated with new imaging modalities; however, physicians should be adequately informed on the potential risks of biological effects. US exposure, according to the ALARA (As Low As Reasonably Achievable) principle, should be as low as reasonably possible.

## 1. Introduction

The first application of ultrasound (US) was performed during World War I for the detection of submarines (sonar) [1]. Since then, US has been successfully used for a wide range of medical and non-medical applications.

In industrial applications, low-power USs with high frequencies are employed to test and measure materials and detect cracks, fractures, moving components, and defects in objects. High-power USs (lower frequencies and higher power) are used for cleaning, welding plastics and metals, cutting and forming materials, separating, mixing, de-gassing, atomizing, localizing, and many others processes [2]. In the food industry, ultrasound technology is generally used in the processing, chilling, and preservation of food products because it is a clean and efficient technique. Ultrasound can be used as an alternative method to thermal treatments for the elimination of microorganisms and enzymes without destroying the nutrients in foods [3]. Another area of ultrasound use is the field of sonobioreactors, which use sound waves to increase the metabolic productivity of microbes, plants, and animal cells [4]. New, promising fields of ultrasound applications include environmental protection and technological procedures to meet industry demands, such as prevention or elimination of existing pollution, water purification, atmospheric decontamination, and soil remediation [5].

In the last decades, there has been extraordinary growth in the use of US imaging as a diagnostic tool in medicine. This has led to the development of sophisticated and powerful modern diagnostic equipment, with significant improvements in resolution, image quality, and gray-scale definition [6]. Nowadays, medical US is used across all medical disciplines (Table 1).

The main advantages of diagnostic US in medicine include real-time assessment, absence of radiation, reduced cost, and portability. Additionally, it can be safely and successfully used to perform biopsies and fine-needle aspirations in parenchymal tissues, such as the lung, kidney, liver, breast, and lymph nodes [7].

At the same time, medical US has also seen development as a therapeutic modality in which energy is deposited in tissue to induce various biological effects [8,9]. Some examples include the use, in surgery, of high-power US for the thermal ablation of tissues and US-guided extracorporeal shock wave lithotripsy (ESWL), which uses high-energy shock waves to break up large kidney and ureteral stones so that stone fragments can be eliminated through the urinary tract. The use of shock waves to treat gallbladder stones has been explored, but the technique has not achieved widespread usage [9]. In dentistry, high-frequency sonic vibrations are used to break apart and remove plaque and tartar during teeth cleaning. High-intensity focused US (HIFU) procedures are used to treat cancer patients. By focusing high-power US onto a target area, the temperature is raised enough to destroy the target tissue. More moderate US power levels are used in physical therapy and sports medicine to deliver moderate heat directly to damaged tissues and to increase and improve the healing of tears, strains, and bruises. Finally, microbubble-based therapeutic strategies are under study for US-directed and targeted therapy. In these strategies, external US exposure activates microbubbles in the circulation, which may act as drug carriers at a desired treatment site [9].

In 1917, Paul Langevin observed for the first time that exposure to high-intensity US was able to kill fish placed in a small tank immediately. Later, some animal studies suggested that prolonged exposure to ultrasound waves could damage neurological, immunological, and hematological systems, as well as the genetic code of the fetus [10]. On the contrary, studies investigating the potentially harmful effects of diagnostic US on human tissues and organs are few, and those available did not show any effect [11]. Based on the currently available data, there is a generally accepted view that the diagnostic use of US is harmless for humans [12]. However, the current state of knowledge does not answer the question of whether there are no side effects associated with ultrasound propagation in human tissues. Although in vitro studies indicate the types of biophysical interactions that may occur and the nature of cellular responses, cell culture conditions may not reflect the in vivo situation. Animal studies are more directly relevant, but transposition between species may be problematic [13]. Given the multiple uses and the exponential development of ultrasound in medical fields, understanding the mechanisms of interaction between ultrasound and biological tissues and their effects on living systems is mandatory.

The purpose of this review was to understand the possible physical interactions of diagnostic ultrasound with biological tissues and the underlying principles for the assumption of the “safety” of acoustic outputs and indices. Observations from in vitro and in vivo studies on the embryo/fetus and specific tissues/organs, even if deriving from outputs exposures above those used in diagnostic ultrasound, have also been reviewed to provide insight into possible bio-effects of ultrasound for the practicing clinician.

## 2. Ultrasound Physics

The term “ultrasound” refers to all acoustic energy with a frequency above human hearing (20,000 hertz or 20 kilohertz). Unlike electromagnetic radiation, which is capable of spreading even in a vacuum, US (as well as sound waves) requires a physical support medium to propagate. US consists of a series of mechanical waves originating from the vibrations of an elastic body that propagate, generally longitudinally, by alternating phases of compression (high density or pressure) with phases of rarefaction (low density or pressure) of the atoms and molecules that constitute the physical medium crossed (solid, liquid, or gaseous).

Like any other type of wave phenomenon, US can be described by its physical properties such as:

- **Frequency (f):** indicates the number of compression and rarefaction cycles (oscillations) of the medium particles that occur in one second. Its unit of measurement in acoustic is Hertz, which corresponds to one oscillation (or cycle) per second (s): 1 hertz (Hz) = 1 (cycle) x s. In diagnostic ultrasound, sound frequencies from 1 to 10 MHz are normally used.
- **Period (T):** represents the time that a wave takes to complete a complete cycle, i.e., the duration of an oscillation. It is measured in seconds (s). Therefore, the period will be shorter the higher the frequency:

(1) $$T=\frac{1}{f}$$

As the period is inversely related to frequency, “f” can also be expressed as 1/s.

- **Wavelength (λ):** represents the space traveled by an oscillation in the time interval of a period or even the minimum distance between two points in which the displacement from the equilibrium configuration assumes the same value (i.e., the distance between two peaks or two troughs of the wave). It is expressed in meters (m).
- **Longitudinal wave velocity (v):** consists of the speed at which sound waves propagate within a physical medium. Frequency, “f”, wavelength, “λ”, and longitudinal wave velocity, “v”, are linked together by the formula:

v = f × λ(2)

Expressing “f” in 1/s and “λ” in m, “v” is expressed in m/s.

Frequency and wavelength are inversely proportional to each other. As the frequency changes, the wavelength automatically changes as well.

The transducers (or probes) used in diagnostic ultrasound are different and work at different central frequencies depending on the type (Table 2). The frequency used influences the theoretical limit of the spatial resolution of the probe (i.e., the ability to discriminate between two distinct but close targets). The higher the frequency, the better the image resolution, but the smaller the depth; conversely, the lower the frequency, the greater the depth; however, the image resolution is reduced. In US equipment, the frequency is selected automatically with the choice of the probe or, in the case of multi-frequency probes, with the choice of a specific pre-set.

The longitudinal wave velocity “v” of the ultrasound is also inversely proportional to the density, “ρ”, and directly proportional to the elastic modulus (or stiffness), “E”, of the transmission material medium, according to the following relationship:

(3) $$v=\sqrt{\frac{E}{\rho }}$$

where “E” is expressed in kg/ms^2^ and “ρ” is expressed in kg/m^3^.

As a result, the longitudinal wave speed of US is modest in gases, while it becomes progressively greater in solids and liquids. The longitudinal wave velocity of US in the air at room temperature is around 343 m/s, while the longitudinal wave velocity in a liquid medium such as water is approximately 1480 m/s. In most human soft tissues, the longitudinal wave speed varies between 1500 and 1600 m/s, with an average longitudinal wave velocity of about 1540 m/s (which is the value on which all US machines used in diagnostics are normally calibrated); adipose tissue is at the lower limits of this range, while muscle tissue is at the higher limits. In bone tissue, longitudinal wave velocity shows values 2–3 times higher than in most soft tissues. The slowest longitudinal wave velocity of US is recorded in lung tissue due to the air content of the alveoli.

- **Acoustic impedance (Z):** consists of the resistance that a material (or tissue) opposes to the propagation of US waves through itself. It is expressed by the product of the density of the crossed medium, “ρ”, by the propagation velocity of US in the medium itself, “v”:

Z = ρ × v(4)

As “ρ” is expressed in Kg/m^3^ and “v” is expressed in m/s, Z is expressed in Kg/m^2^s. Human tissues are not homogeneous in terms of composition. Table 3 shows approximate densities, acoustic impedances, and US longitudinal wave velocities for a variety of biological tissues [14,15,16,17].

The amount of change in acoustic impedance encountered by the US beam as it passes through biological tissues will determine the amount of US signal that will be reflected (i.e., come back towards the transducer), refracted (i.e., deflected from a straight path), dispersed from the microscopic inhomogeneities of tissues (i.e., scattering), or absorbed.

- Amplitude, acoustic power, and intensity are quantities that variously define the mechanical energy carried by the US. **Amplitude (A)** defines the maximum compression peak of the wave, and it is generally expressed in Pascal (Pa). **Acoustic power (P)** is the amount of sound energy carried in the unit of time, and it is expressed in Watts (W). **Intensity (I)** is a measure of acoustic power per unit area and, thus, the amount of sound energy delivered to biological tissues. It is expressed in W/cm^2^. As it will be shown, intensity determines the biological effects of US, and its values are regulated on the basis of parameters established by competent governmental agencies at the national and international levels. In B-Mode applications, the acoustic power of the transducer ranges from 10 to 18 mW. In M-Mode applications, the transmitted acoustic power is less than 4 mW, while in Doppler and color-Doppler applications, the transmitted acoustic power is 30 mW and 80 mW, respectively. Alternatively, in acoustics, the intensity level of a sound may be expressed in decibels (dB), defined as the logarithmic ratio of the intensity, “I”, with respect to a reference sound intensity, “I_0_”, which coincides with that of the lowest sound perceivable by the human ear: dB = 10 Log (I/I_0_).

## 3. Quantifying Biological Effects

The need to identify a physical quantity that can be directly related to the biological response is an essential prerequisite for understanding the potential risks of ultrasound interaction with biological tissues in controlled experimental studies [13]. The most widely used quantity in the study of the bioeffects of US is the intensity expressed in W/cm^2^. Regulatory authorities for the use of diagnostic US mainly consider the spatial-peak temporal-average intensity (I_SPTA_), which is the maximum intensity in the beam averaged over the pulse repetition period (PRP). This is because the higher the “pulse on” time, the greater the acoustic energy delivered to biological tissues. However, this does not provide a real measure of the absorbed “dose”. Indeed, the distribution of acoustic energy across the scanned area varies from point to point, also depending on the duration of the examination, the acoustic impedance of the organs examined, and the penetration capacity of the US beam. As a result, the measure of acoustic power alone incompletely describes tissue exposure and the potential biological danger.

Two basic mechanisms of US interaction with biological tissues have been identified: thermal and non-thermal. Usually, both effects occur simultaneously but with different intensities.

In 1992, in a joint conference between the American Institute of Ultrasound in Medicine (AIUM) and the National Electrical Manufacturers Association (NEMA) (AIUM/NEMA, 1992 [18]), two standard indicators of acoustic power and biological risk from ultrasound were defined: the Mechanical Index (MI) and the Thermal index (TI).

The **thermal index (TI)** is the indicator of the potential temperature increase resulting from the friction of interfaces stressed by compression and rarefaction waves.

The **mechanical index (MI)** is the indicator of potential non-thermal mechanical effects of cavitation determined by the negative pressure peak or rarefaction of the US beam.

The same 1992 AIUM/NEMA joint conference also first defined and published the so-called “Output Display Standard” (ODS), in which it was mandated that diagnostic US machines be able to display on the screen a thermal index and a mechanical index as safety indices to standardize diagnostic US examinations and provide information to the user related to safety [18].

In the USA, the Food and Drug Administration (FDA) developed guidelines on acoustic output levels considered acceptable and demanded that the ODS information be provided by the manufacturers [19], while in Europe, ultrasound imaging scanners are required to meet the standard requirements for safety and effectiveness set by the International Electrotechnical Commission (IEC) to be sold. IEC standards for diagnostic and monitoring equipment (IEC 60601-2-37:2007+AMD1:2015 CSV [20]) set no upper limit on output exposure quantities but specified methods for the determination of TI and MI under a set of particular conditions and explained how a user should be informed about potential hazard through its displayed values.

## 4. Thermal Effects

As the propagation of the US energy, and thus, the distance traveled in a material (e.g., a tissue) increases, there is a corresponding decrease in the amplitude of the wave. This is due to absorption and/or dispersion (scattering) of the US signal. Absorption is the portion of the wave energy converted into heat energy in the biological structures traversed by the US beam; dispersion is the portion of the wave that changes direction. As the tissue can absorb energy to produce heat, an increase in temperature may occur. The increase in temperature will be as greater as the rate of ultrasound heat production exceeds dissipation.

Assuming the case of an US beam passing through an absorbing target, the energy deposited as heat, “Q”, in an ultrasound field of intensity, “I”, is given using the following equation [21]:

Q = αI(5)

where “α” is the absorption coefficient depending on various media or tissues.

As a result, an increase in the input intensity (or in the power) of the US beam will result in an increased potential for tissue heating. In addition, since the acoustic intensity describes the acoustic power passing through a unit area of tissue, the smaller the scanned area, the greater the energy passing through it. This will also result in a greater amount of heat deposited. Powerful transducers and strongly focused fields, therefore, will produce particularly significant temperature increases. On the other hand, intensity and heat production decrease when ultrasound energy is distributed over a larger area (unfocused). However, the condition described is an oversimplification. In modern ultrasound scanners, where a focused imaging beam typically originates from more than 100 array elements, the intensity at the focus is much higher than that at the input. Furthermore, over the past decade, improvements in transducer design and manufacturing techniques have led to two-dimensional matrix arrays and multi-focal imaging beams, particularly found in four-dimensional (4D) fetal ultrasound scanners, greatly increasing the ultrasound intensity delivered to the imaged volume.

Deposited heat increases linearly with the absorption of the medium. In general, the concentration of proteins increases the absorption of ultrasound, so tissues with greater collagen content absorb more energy. Attenuation coefficients have been measured for a range of biological tissues in vitro and in animal studies and are available in the literature [14,15,17,22,23]. (Table 4). However, caution needs to be exercised in using some of the published thermal property values for tissues, in particular for values obtained in vitro and from post-mortem studies. Indeed, heat transfer in biological media is affected by manifold in vivo conditions, including the effect of blood perfusion and heat generation due to metabolism.

Although attenuation includes the small additional contribution from the scattering of an ultrasound beam by tissue, the contribution of absorption to attenuation may be 60–80% of the total [23]. Therefore, in tissues such as skeletal muscle or even bone—which are characterized by a higher attenuation coefficient and, thus, absorption—we will expect a greater increase in temperature than, for example, in adipose tissue.

In addition, the absorption coefficient, “α”, is a function of frequency, “f”, of the ultrasound energy, according to the following relation [22]:

α = af^b^ (6)

where a and b are tissue-specific constants.

As stated above, higher acoustic frequency waves display better spatial resolution, but they imply less beam penetration depth. Additionally, as the highest frequency beams are also absorbed more strongly, they have the potential to produce heating in superficial tissue.

Increasing the time that any particular region is exposed to an US beam (dwell time) may increase the temperature rise produced. For a given US field of intensity, “I”, assuming that no heat is lost by conduction, convection, or any other heat removal processes, the energy deposited as heat, “Q”, is approximately described by the equation formulated by Fry and Fry in 1953 [24]:

(7) $$Q=\rho c\frac{\delta T}{\delta t}$$

where “ρ” represents the tissue density, “c” is the specific heat of the tissue (i.e., the amount of heat required to increase the temperature of tissue by 1 °C per unit of mass), “δT” is the local temperature increase, and “δt” is the time duration of exposure.

Equation (7) is valid only for short exposure times. For longer exposure times, heat removal processes become significant.

As noted above, in living tissue, heat transfer depends partly on the rate of heat conduction due to temperature gradient, partly on the rate at which the heat is removed by blood flow (i.e., blood perfusion), and partly on the thermal contribution due to the metabolism. Each of these mechanisms is taken into account in the partial derivative Pennes bio-heat equation [25], which is given by:

(8) $$\rho c\frac{\delta T}{\delta t}=k\;\left(\frac{\delta^{2}T}{{\delta x}^{2}}+\frac{\delta^{2}T}{{\delta y}^{2}}+\frac{\delta^{2}T}{{\delta z}^{2}}\right)+\omega_{b}c_{b}(T_{a}-T)+Q_{\mathrm{met}}$$

where “k” is the tissue thermal conductivity (i.e., the tissue capability to conduct heat), while “δx”, “δy”, and “δz” describe the change in the temperature, “T”, over the directions x, y, and z. ω_b_c_b_(T_a_ − T) is the blood perfusion term, where “ω_b_” is the mass flow rate of blood per unit volume, “c_b_” represents the specific heat of the blood, and T_a_ is the temperature of the arterial blood. “Q_met_” represents the metabolic heat generated per volume unit of tissue.

Although the Pennes equation configures a simplified bio-heat transfer model in which the metabolic heat generation and the blood perfusion effect are assumed to be homogeneously distributed, it provides the best mathematical description for the propagation of thermal energy through living tissues. In general, well-perfused tissues are less susceptible to US-induced temperature rise because local perfusion, mainly by convection, allows some of the produced heat to be transported away from the site of generation. On the contrary, poorly perfused biological tissues, such as lens, cornea, tendon, and adipose tissue, may be particularly susceptible to the thermal effects of US. Similar concerns have been raised about early gestation because of the lack of or the minimal perfusion of the first-trimester embryo. Moreover, cells are more susceptible to external stimuli during periods of rapid division, such as embryogenesis. For these reasons, the embryo/fetus should be considered to be at risk of thermal effects, and operators should attempt to ensure that the exposure is well managed [26].

Absorption of US, together with losses due to dispersion, reflection, and refraction, leads to a greater reduction in ultrasonic intensity in the propagation path. Therefore, US beam intensity is greatest near interfaces, and maximum temperature shifts closer to these passage points. The presence of bone within the US path greatly increases the likelihood of thermal effects for adjacent tissues because of bone’s high absorption coefficient and heat conduction from bone itself to interface structures. The very high attenuation and absorption coefficients exhibited by lung tissue can be explained by the absorption and radiation of acoustic energy from gas bubbles enclosed in alveoli. This should imply caution for eventual thermal effects [14].

Finally, different machine settings may affect heating through their effect on the acoustic power or the beam area. Scanned modes such as B-mode real-time imaging and color-flow Doppler distribute the beam’s energy over a wide area, whereas unscanned modes such as spectral Doppler and M-mode concentrate the energy along a stationary line, resulting in potentially increased temperatures. Similarly, continuous-wave US concentrates more energy in tissue than the on/off cycle of pulsed wave US [26].

## 5. The Thermal Index

The thermal index (TI) provides information about the increase in tissue temperature.

The TI is defined as the ratio between the acoustic power output from the ultrasonic transducer (anywhere in the US field), “P_0_”, and the acoustic power required to increase tissue temperature of 1 °C, contributed by US absorption alone under specific and conservative conditions, “P_deg_” (ODS, 2004 [18]):

(9) $$\mathrm{TI}=\frac{P_{0}}{P_{\deg}}$$

In biological models, a TI = 1 corresponds to 1 centigrade degree (°C) of increase in temperature for a given transmission power, probe frequency, scan area, and exposure time, considering the attenuation and absorption characteristics of biological structures. It should be stressed that the displayed TI is not the actual temperature increase in °C generated in tissue while scanning. TI is, however, the best indication of thermal hazard available to the user and allows risk to be quickly assessed during an US examination [12].

In the USA, where the acoustic output levels are limited by the FDA, the higher-output devices are allowed to produce a maximum ISPTA of 720 mW/cm^2^ for all applications. US is not recommended when the TI is greater than 6.0. An exception is related to ophthalmic application, for which the FDA limits the ISPTA to 50 mW/cm^2^ and does not recommend US examination when the TI is greater than 1.0 [19,27]. The lower limits applied for eye scanning reflect concerns that the eye may be particularly susceptible to thermal damage as a result of very low blood perfusion.

Regular cellular activity depends on chemical reactions occurring at a certain rate. The rates of chemical reactions and, thus, enzyme activity depend on temperature. An immediate consequence of an increase in temperature is an increase in the speed of biochemical reactions. However, when the temperature becomes sufficiently high, enzymes become denatured. Subsequently, enzyme activity decreases and eventually ceases, which can have a significant impact on the structure and function of cells. At higher temperatures, protein coagulation may occur. Harmful effects in vitro are generally noted at temperatures of 39–43 °C if kept for a sufficient period of time [28].

Depending on US intensity, a particular exposure period is required before the tissue reaches a harmful temperature increase. The British Medical Ultrasound Society (BMUS) guidelines for the safe use of diagnostic US equipment [29] recommend limiting exposure time at higher TI values.

Starting from theoretical (Jago et al. 1999 [30]) and experimental (Shaw et al. 1998 [31]) studies, BMUS guidelines stated that, in some circumstances, TI could underestimate the temperature elevation by a factor of up to two. For example, a TI value of 1 is considered to correspond to a worst-case temperature elevation of 2 °C.

In adult tissue, for temperature increases less than or equal to 2 °C above normal (i.e., 37 °C), there have been no significant adverse biological effects observed for durations of temperature elevation up to 50 h.

For temperature increases more than 2 °C above normal (1.0 < TI ≤ 6.0), scanning times have to be limited, as follows:

- 1.0 < TI ≤ 1.5: 120 min;
- 1.5 < TI ≤ 2.0: 60 min;
- 2.0 < TI ≤ 2.5: 15 min;
- 2.5 < TI ≤ 3.0: 4 min;
- 3.0 < TI ≤ 4.0: 1 min;
- 4.0 < TI ≤ 5.0: 15 s;
- 5.0 < TI ≤ 6.0: 5 s;
- TIB > 6: not recommended.

Three different thermal indices were developed to address three different tissue models, namely the Thermal Index of Soft Tissue (TIS), the Thermal Index of Bone (TIB), and the Thermal Index of Cranial bone (TIC).

1. Thermal Index of Soft tissue (TIS): The soft tissue model assumes a uniform homogeneous soft tissue beam path. There is no bone, developing bone, or cartilage anywhere in the region being scanned.
2. Thermal Index of Bone (TIB): This model assumes a layer of strongly absorbing material (bone) within the soft tissue model.
3. Thermal Index of Cranial bone (TIC): This model omits soft tissue and considers the absorption of ultrasound in a bone layer located directly under the transducer.

These three different tissue models can be used to estimate TI in two different scan modes, namely the scanned mode and unscanned mode, resulting in six combination conditions.

The scanned mode is associated with pulsed B-mode ultrasound and Doppler imaging of cross-sections of tissue. The unscanned mode is typically used clinically for M-mode and spectral Doppler studies. The need for two exposure conditions arises from the different geometric distributions of acoustic power; the unscanned mode concentrates the power of the ultrasound beam on a stationary focal region, which can be as narrow as 1 mm^2^. In scanned mode, the acoustic power extends over a much larger region of exposed tissue, typically covering 100 mm^2^ or more. The IEC standard specifies how to derive the TI value under each of the six circumstances described using measurements of acoustic quantities at prescribed positions within the beam [32].

### 5.1. Thermal Index of Soft Tissue (TIS)

The Soft Tissue Thermal Index (TIS) provides information on the temperature rise in areas in which only soft tissues are scanned, such as in abdominal examination or during obstetric scanning earlier than 10 weeks of gestation. The soft tissue model assumes a uniform homogeneous medium with an attenuation coefficient of 0.3 dB/cmMHz. Actually, the average attenuation coefficient for fatty and non-fatty soft tissues is about 0.4 and 0.6 dB/cmMHz, respectively [32]. The use of an absorption coefficient somewhat lower than soft tissue in this model allows us to safely assume the inclusion in the propagation path of areas in which the ultrasonic pulse passes through a fluid space, such as amniotic fluid or urine (where the attenuation coefficient is much lower than that of soft tissues). The soft tissue model also allows us to make assumptions about heat loss from blood perfusion.

### 5.2. Thermal Index of Bone (TIB)

The Thermal Bone Index provides information on the increase in temperature in or around strongly absorbing bone structures within the soft-tissue model. In this model, US passes through a homogeneous tissue and then reflects off the plane of the bone perpendicular to the beam. If the bone is within the focus, then the temperature in the bone will increase. Examples of the application of TIB are the second and third trimesters of fetal imaging, adult thoracic examination, and musculoskeletal scan of the upper and lower limbs. The attenuation and absorption coefficients of the layer are not defined [32]. However, the formula assumes that half of the incident power is absorbed in this model.

### 5.3. Cranial Bone Thermal Index (TIC)

The Cranial Bone Thermal Index (TIC) assumes a near-surface bone model. In this case, all the US power is assumed to be absorbed by the bone, which is coupled directly to the transducer, omitting the presence of soft tissue [12,32].

## 6. Acoustic Cavitation Effects

The concept of cavitation refers to phenomena related to the vibrations and motion dynamics of gas microbubbles located in an ultrasonic field.

For cavitation to occur, gas bubbles or nucleation sites within the fluid or tissue are required. There is no evidence for the presence in vivo of cavitation nuclei that may be excited by diagnostic ultrasound. Viscous and other forces within solid tissues seem to make the probability of cavitation events quite small [33]. On the contrary, cavitation bubble formation has been observed with very high amplitude ultrasound pulses used in lithotripsy, where bubble collapse can generate high-speed fluid microjets used in stone fragmentation [34]. Gas-containing tissues, such as the lung and the intestine, may be more vulnerable to damage from diagnostic exposures than apparently gas-free tissues [35]. Furthermore, the introduction of gas microbubbles used as US contrast agents into the body by intravenous injection significantly increases the potential for cavitation during clinical ultrasound examinations.

When a fluid with microbubbles is exposed to an acoustic field, gas-filled microbubbles undergo oscillating changes in volume due to the acoustic wave. They expand in size during the period of decreased pressure and contract during compression to an extent dependent on the acoustic pressure. This phenomenon is known as cavitation [26].

Two types of cavitation phenomena exist, non-inertial cavitation and inertial cavitation [34].

Non-inertial cavitation, sometimes also known as “stable” cavitation, describes a repetitive oscillation around the equilibrium radius of a bubble in a liquid exposed to an acoustic field without large changes in volume. The maximum expansion of a gas microbubble in non-inertial cavitation typically does not exceed more than two times the equilibrium radius. However, a variety of non-linear physical phenomena can be associated with non-inertial cavitation; bubble oscillations can result in heat generation, microflow of fluid near the bubble, and localized shear forces (shear stress).

In inertial cavitation, sometimes called “collapse cavitation”, the bubble can expand more than twice its initial radius and then rapidly collapse. The collapse may generate a strong shock wave, which is accompanied by extremely high local temperature values that are associated with the release of free radicals. Free radicals can cause undesirable biological effects, such as biochemical reactions between tissues.

According to the theoretical model postulated by Holland and Apfel in 1989 [36], the initial size of the cavitation nuclei determines the minimum acoustic pressure and the optimal frequency required for significant bubble growth. An initially smaller-sized microbubble needs a higher acoustic pressure amplitude to overcome the stronger surface tension. Higher frequencies require a very specific and small bubble radius for cavitation. At lower acoustic pressure, oscillations in bubble size occur broadly according to variations in acoustic pressure in a stable fashion, making stable cavitation more likely. However, if the peak acoustic pressure increases, different motions may be induced until, finally, the bubble becomes unstable and collapses under the inertia of the surrounding liquid. Higher frequencies shorten the interval between the compressive phases of the US wave, thus reducing the growth of gas-filled bubbles. On the other hand, lower frequencies increase the probability that the microbubble will collapse.

At the MHz frequencies used in diagnostic ultrasound (i.e., 1–10 MHz), the most efficiently echogenic (i.e., resonant) pre-existing gas bodies have to be few in diameter (i.e., about 1–5 µm) [36]. However, for diagnostic ultrasound, which operates at higher acoustic frequencies and lower pressure amplitudes than lithotripsy, the possibility of gas body activation and inertial cavitation in solid tissues is likely null. Church [33] reported that the threshold pressure for acoustic cavitation to occur within soft tissue without pre-existing nuclei is greater than 4 MPa at 1 MHz for a 1 ms pulse and, therefore, is above those used in current diagnostic practice.

Cavitation bioeffects have been observed in tissues naturally containing gas pockets in vivo. Pulsed ultrasound within the range of parameters of diagnostic ultrasound was found to induce lung and intestinal hemorrhage in animal models, while such effects have never been reported in humans [37]. Anatomical structural differences among species and a different distribution of gaseous cavitation sites have been suggested to influence the susceptibility to pulsed ultrasound [11]. However, the potential risk of harm to patients is still not clearly understood, and caution is required in clinical practice.

Holland et al. [38] reported that US contrast agents could significantly lower the threshold required for acoustic cavitation. Ultrasound contrast agents consist of a suspension of gas-filled microbubbles, typically ranging in size from less than 1 to 10 µm in diameter. The non-linear behavior of pulsing microbubbles has a key role in their effectiveness as US contrast agents. When pulsing, the bubbles send secondary sound waves in all directions. These secondary sound waves enhance US images because they also reflect back to the transducer. However, microbubble compression cycles with negative and positive pressures also result in secondary motion, high shear stress or local shear forces, and microstreaming of the surrounding fluid. These events may result in the fragmentation of subcellular and cellular structures. Furthermore, the gas bodies can destabilize and act as inertial cavitation nuclei. Reported bioeffects following US contrast microbubble injection and US exposure in vivo include hemolysis, damage to the microvasculature, glomerular capillary hemorrhage, the opening of the blood–brain barrier, inflammatory cell infiltration, cardiomyocyte death, and effects on cardiac rhythm [39,40]. These effects are most likely to arise from the destruction of the microbubbles [41]. Over the years, advanced and more stabilized contrast microbubbles have been designed to improve persistence in the circulation, and new ultrasound imaging modes have been developed specifically for imaging contrast agent gas bodies [42]. Prudent use of diagnostic ultrasound with contrast agents is always recommended, particularly in patients with a history of myocardial infarction or unstable cardiovascular disease [43].

## 7. Mechanical Index (MI)

The mechanical index (MI) is an indication of an US beam’s ability to cause cavitation-related bioeffects.

This index is based on the theoretical model assumed by Apfel and Holland in 1991 [44], who, describing the behavior of a cloud of air bubbles of a wide range of diameters in water and blood, determined the approximate acoustic pressure amplitude required to cause a bubble to undergo inertial cavitation (i.e., a large expansion followed by a rapid and violent collapse).

The MI is defined as the estimated peak rarefactional pressure “P_r_” (anywhere in the US field) adjusted for tissue attenuation “α”, divided by the square root of the center frequency “f” of the US beam [44]:

(10) $$\mathrm{MI}=\frac{\mathrm{Pr}\alpha }{\sqrt{f}}$$

MI allows the acoustic pressure threshold necessary to cause cavitation and, thus, potential damage, to be identified.

The threshold pressure for acoustic cavitation is above that used in diagnostic ultrasound. In HIFU lithotripsy, the peak acoustic pressure is typically in the order of 10–100 MPa compared to approximately 1 MPa for diagnostic ultrasound, and the acoustic frequency of HIFU pulses is much lower than diagnostic pulses. These factors make the eventual tiny cavitation nuclei present in all liquids and tissues more likely to become activated and cavitate. The FDA approved a MI value of 1.9 as the maximum threshold for diagnostic imaging [19]. Apfel and Holland [44] showed that the minimum threshold for inertial cavitation of optimally sized nuclei is roughly near a MI value of 0.5 in blood conditions. However, gas bodies suitable for nucleation of ultrasonic cavitation (i.e., about 1–5 µm) are normally unlikely to exist in the human body (due to surface tension and rapid dissolution of the gas), minimizing the possibility of cavitational bioeffects for diagnostic ultrasound. BMUS set a MI value of 0.7 as the threshold for cavitation if an ultrasound contrast agent containing gas microspheres is being used [29]. The American Institute of Ultrasound in Medicine issued an official statement informing the clinical ultrasound community of the potential for bioeffects from diagnostic ultrasound with US contrast agents for MI values above about 0.4 [43].

## 8. Other Non-Thermal Mechanical Effects

In addition to heating and cavitation, US has the potential to induce other bioeffects by non-thermal mechanisms. These secondary effects tend to increase with increasing intensity and are generally relatively small. However, due to the essential non-linearity of the acoustic equations, they have the potential to produce forces and motions at frequencies much lower than those of incident ultrasonic waves [45]. Typically, when an object is placed in the path of the ultrasound beam, a force acts upon it. This force, also called acoustic radiation force (ARF), develops in the direction of the US wave propagation and results in a transfer of energy from the US field to the object. The magnitude of the ARF depends on the characteristics of both the US field and the object within it. Obviously, ARF is most relevant for structures that are free to move, such as fluid particles; solid tissue is only slightly compressed.

For a plane wave incident normally on an object, it can be expressed using the following equation [45]:

(11) $$\mathrm{ARF}=\frac{2\alpha I}{v}$$

where “α” is the absorption coefficient of the object, “I” is the average temporal intensity of the acoustic wave (W/cm^2^), and “v” is the propagation speed of US in the medium (cm/s).

For the case of a plane wave normally incident on a perfectly absorbing target, the radiation force is equal to I/v. On the other hand, the ARF normally exerted by a plane wave on a perfectly reflecting target is twice that of a perfectly absorbing target since the wave now also travels in the opposite direction. For partly absorbent and partly reflecting interfaces, the ARF varies between I/v and 2I/v. These plane wave approximations provide adequate estimates of the ARF [34]. In the case of a continuous wave signal, the ARF will be a constant pressure. If, however, the acoustic signal is pulsed or modulated, the ARF will vary periodically at the pulse or modulation frequency.

ARF is the mechanism behind phenomena such as radiation momentum and acoustic streaming.

If US radiation pulses are transmitted into a tissue, the radiation momentum of the ultrasound waves will be transferred to the tissue, and it will be pushed on in the direction of ultrasound propagation. When the ARF ceases, the tissue will return to its equilibrium position. When this occurs, waves of axially polarized motion will propagate transverse to the direction of the US ARF application [34]. These displacements can be monitored both spatially and temporally and are called shear waves. The shear wave velocity (SWV) is proportional to the elastic characteristics of the tissue being examined. This phenomenon is utilized in ultrasonic elastography using Acoustic Radiation Force Impulse (ARFI) imaging.

An acoustic field propagating in a fluid medium can give rise to a flow called “acoustic streaming” [46]. When a continuous sinusoidal ultrasound wave propagates through a fluid, it forces the fluid particles to oscillate sinusoidally in the wave propagation direction. In linear acoustics, it is assumed that the wave shape does not change over time and that particles in the medium simply vibrate around their equilibrium position. Consequently, no net flow is assumed. However, the attenuation of an ultrasonic beam with distance can be considered as creating a “radiation pressure gradient” in the medium. As a result of such a gradient, each fluid element experiences a net body force that gives rise to a net flow. Typically, the flow may be away from the transducer axis, but recirculation vortices may be created that bring the fluid back toward the transducer surface [45]. The trajectory of fluid particles will be governed by the balance between the viscous drag force and the acoustic radiation force [34]. The phenomenon of acoustic streaming can assist in the diagnosis of fluid-filled cysts versus solid tumors using the pulsed-Doppler mode to produce and observe the fluid motion [47].

Another important mechanical action of US is the phenomenon of so-called “acoustic microstreaming”. Acoustic microstreaming specifically refers to the streaming flow of fluid around an oscillating gas microbubble [46]. When microbubbles are close to endothelial cells, such microstreaming may result in shear stress of the endothelium and changes in membrane permeability [32]. One acute effect of endothelium shear stress (occurring from seconds to minutes) is flow-dependent dilation. This effect relies on factors secreted by the endothelium, such as nitric oxide (NO). However, shear stress also modulates the formation of superoxide anions, whose excessive production has been shown to be harmful to the membrane integrity of the cell [48,49]. Acoustic microstreaming was also postulated as a direct mechanism by which ultrasound causes changes in membrane permeability, and this action may result from a mechanical effect on ion channels [49]. A further indirect effect of endothelium shear stress is platelet “activation” with the consequent production of pro-coagulant and pro-inflammatory mediators [50]. The effect of microstreaming has been confirmed with microbubbles injected into the vascular system in the form of contrast media [51]. The average distance of microbubbles from the endothelium increases with vessel size, and the effects of microstreaming may be dominant in the capillary bed. However, a damaged endothelium can lead to the attachment of microbubbles even in large vessels [49]. Lindner et al. [52] observed prolonged dwell time of Albunex microbubbles in sites where the endothelial glycocalyx was damaged. Yasu et al. [53] observed the retention of microbubbles in inflamed venules. In therapeutic or experimental applications, disruption of endothelial integrity may be intentional, such as for the delivery of genetic material or drugs [53,54]. However, for the diagnostic application of microbubbles as contrast agents, endothelial disruption is clearly undesirable [49].

## 9. Strengths and Limitations in the Use of TI and MI Indices in Clinical Practice and Further Safety Recommendations

Safety index values displayed on the monitor can give very valuable information, previously hidden from the user, about the potential mechanical and thermal risks associated with each US examination. Typically, the values of MI and TI alter dynamically as the practitioner changes the mode of operation or output power. For example, higher TI values may be shown when the mode of operation of the scanner is changed from conventional B-mode imaging to Doppler-mode imaging or when a deeper focal zone or a smaller field of view is selected. Higher MI values may be expected when harmonic imaging is used.

However, the use of the above-presented indices has some limitations that need to be considered. First, TI and MI indices are mainly related to the transposition of tissue model assumptions to the human being. They may be under- or over-estimated in poorly or highly perfused tissues and do not take into account inter-human variability. The TI and MI indices do not consider either the scanning time, the possibility of non-linear effects, or the self-heating of the transducer that could lead to an increase in the surface temperature of the scanned area. Thermal indices are steady-state estimates and may not be appropriate for new imaging techniques [55]. The TI value displayed on the screen does not correlate directly with the actual temperature change. TI and MI values are not valid for US contrast agent applications because contrast agents lower the cavitation threshold. Finally, the method used by the manufacturer to update the index dynamically may use algorithms that are not specified by the ODS, with resulting errors that can be as large as 100% and should be described in the machine manual [56].

As a consequence, the responsibility for the safe use of diagnostic US has to be transferred to the user. According to the European Federation of Societies for Ultrasound in Medicine and Biology (EFSUMB) [57], US should be used only by qualified health professionals who can quantify the potential benefits and risks to the patient during each type of examination. In addition, both the EFSUMB [57] and the American Institute of Ultrasound in Medicine (AIUM) [58] recommend the following ALARA (As Low As Reasonably Achievable) [59] general principles of prudent use of diagnostic methods based on the interaction of US energy with biological tissues.

1. Apply the presets of an examination correctly if they are integrated into the US diagnostic device. Reviewing the factory default presets to verify their suitability is encouraged.
2. Adjust the power to the lowest setting available to produce diagnostic-quality images. If appropriate, reduce the power at the end of each examination so that the next patient starts at the lowest sound setting.
3. Monitor the mechanical index (MI) and thermal index (TI). Get to know the recommended upper limit of MI, TI, and related duration limitations for the type of examination to be performed.
4. Move/remove the transducer when stationary imaging is not needed so as to reduce the time spent on a particular anatomical structure. Whenever possible, avoid scan fields that include sensitive tissues such as the eye, gas-filled tissues (lung and intestines), and calcified fetal structures (skull and spine).
5. Minimize the overall scan time to obtain the required diagnostic information [27].

## 10. Observation of Biological and Clinical Effects of Ultrasound

In vitro and in vivo animal studies have shown that US can cause a wide range of effects at the molecular, cellular and organ level. Understanding these effects can improve our knowledge on the complex interactions between US and biological tissues, so that new avenues for new diagnostic therapeutic applications can be opened.

### 10.1. Molecular Effects

In 2001, Keychain et al. [60] demonstrated that diagnostic US can greatly enhance endothelial uptake of bioactive proteins in vivo. In vitro experiments on cultured human umbilical vein endothelial cells (HUVECs) have shown that internalization of caveolae without alteration of cell membrane integrity is a novel mechanism of US-induced protein uptake by endothelial cells associated with phosphorylation/activation of ERK1/2 [35,61]. Diagnostic US, with its energy, allows the transition from the latent enzyme-purified human precallicrein into the active enzyme kallikrein [62]. This explains the triggering action of US on the intrinsic human coagulation pathway. As a clinical consequence, patients at risk of thrombosis and patients with liver failure should be protected by low-molecular-weight heparin before US exposure, especially if prolonged.

### 10.2. Cellular Effects

US may be responsible for the production of reactive oxygen species (ROS) that cause cell apoptosis. Andreassi et al. [63] concluded that cardiac US (1–3.6 MHz and MI 1.5) in vitro increases intracellular oxidative stress. The increased production of reactive oxygen species (ROS) was confirmed by morphological evidence of endothelial damage only after longer exposure times (30 s), while US exposure longer than 15 s has been shown to induce significant DNA scattering and a loss of lactate dehydrogenase (LDH). These results were obtained in vitro and, of course, cannot be confirmed in vivo, where various antioxidant systems (i.e., glutathione, ascorbate, catalase) can rapidly inactivate H^.^ and OH^.^ radicals, making the production of ROS due to US negligible [35]. Exposure to US in Doppler mode has been shown to increase antioxidant enzyme activity in rat liver and brain. In contrast, after B-mode (4 MHz) US exposure, antioxidant enzyme activity was decreased in fetal brain tissue due to its higher lipid concentration [64]. In neural cells, heat shock proteins (HSPs) are constitutively expressed and prevent or correct polypeptide folding, thus protecting neurons from injury [65]. A rapid increase in temperature associated with US exposure (30 min at 1.2 W/cm^2^) increases the production of HSPs and may, therefore, produce a neuroprotective effect. Furthermore, the upregulation of HPSs may have an additive therapeutic role in relation to its believed importance in sensitizing tissue to radiotherapy and chemotherapy in non-ablation thermal treatments [66]. When combined with systemic hyperthermia, however, US-induced temperature increases may contribute to the development of congenital malformations in experimental animals [67]. US can also influence cell regeneration. In the study by Tarantal et al. [68], repeated US exposure reduced leucocyte production in monkeys’ uteri. Similarly, a decrease in the number of somites was noted when embryo cultures were exposed to US for 15 min at 40 °C [67]. A non-thermal injury mechanism has been proposed as responsible for these effects.

### 10.3. Genetic Effects

Genetic, chromosomal, and other mutations have been extensively studied as possible consequences of exposure to US waves, but whether these can lead to physiological consequences is still controversial [10]. These effects presumably occur due to the increase in oxygen free radicals and their action on the cellular nuclei.

### 10.4. Fetal Effects

There is no evidence of immediate or long-term harm to a developing fetus from exposure to B-mode US. Available evidence does not support an association between the use of US for fetal imaging with cancer or with adverse effects on birth weight, growth, or neurodevelopment during childhood [69]. Multiple exposures to US in utero was associated with a small increase in the incidence of low birth weight compared to a single exposure, but this difference was not statistically significant and disappeared as the babies grew up [70]. A delay in language development in children exposed to US was also reported, but this difference was not maintained during later development [71].

However, national and international societies continue to urge caution about the use of US in obstetric applications, particularly regarding the possibility of thermal effects [72]. Conditions present in early pregnancy, such as lack of perfusion, may favor US-induced temperature rise. Furthermore, the rapid cellular division occurring during embryogenesis increases the vulnerability of DNA to thermal insults. The teratogenic effect of heat on mammals is well recognized, with the developing central nervous systems exhibiting the greatest sensitivity [73]. In view of these considerations, the World Federation for Ultrasound in Medicine and Biology (WFUMB, 1998 [74]) concluded that an US exposure that elevates human embryonic or fetal temperature by 4 °C above normal for 5 min should be considered potentially hazardous.

In particular, the use of color flow or pulsed Doppler increases the potential temperature rise by an unknown factor and makes thermal indices exceeding three times the recommended limit possible. Indeed, in animal models, Doppler US exposure in utero has been shown to give rise to increased apoptosis [75]. Moreover, the thermal hazard has been greatly increased with the development of US applications in fetal imaging beyond medical practice into commercial souvenir scans, such as 3D imaging systems, that can provide clearer images of fetus anatomy recognizable to the family and 4D sonographic equipment that further facilitates observing fetus movements. In particular, continuous exposure in 4D sonography has the potential to prolong examination times and, thus, increase the risk for bioeffects.

As there is very little information currently available regarding possible subtle biological effects of diagnostic levels of US on the developing human embryo or fetus, care should be taken to limit the TI and MI and the exposure time to the minimum commensurate with an acceptable clinical assessment. In general, US examinations in obstetrics should be as short as possible. Ultrasound scans should not be performed solely for producing souvenir images or recordings of a fetus or embryo. According to BMUS guidelines [29], for obstetric and neonatal scanning, there is no known reason to restrict scanning times with a TI value between 0–0.7. The test time should be reduced if TI > 0.7, as follows:

- 0.7 < TI ≤ 1.0: 60 min;
- 1.0 < TI ≤ 1.5: 30 min;
- 1.5 < TI ≤ 2.0: 15 min;
- 2.0 < TI ≤ 2.5: 4 min;
- 2.5 < TI ≤ 3.0: 1 min;
- TI should never exceed 3.0.

The International Society of Ultrasound in Obstetrics and Gynecology (ISUOG) recommends a TI < 1.0 for first-trimester screening (11 weeks to 13 weeks, 6 days); pulsed Doppler and color Doppler should not be used routinely. If a Doppler test is required, it can be performed maintaining a TI < 1.0 for no more than 5–10 min. Examination of the uterine vessels of the mother is safe when the fetus is outside the irradiated field [72]. Special care should be taken to reduce the risk of thermal and non-thermal effects during cardiac, pulmonary, and cranial investigations of neonates. As there is experimental evidence that self-heating of the transducer can lead to a significant increase in skin surface temperature, scan times and exposure levels should be kept as low as possible. For neonatal ultrasound, there is a potential risk of lung damage; for MI > 0.7, the risk of cavitation increases, especially in studies with contrast media. The exposure time should be shortened if the lung or intestine is scanned at MI values above 0.3 [29]. When attempting to obtain fetal heart rate with a diagnostic US system, the AIUM recommends using M-mode [76]. The power levels used for fetal heart rate monitoring (cardiotocography—CTG) are sufficiently low, so the use of this mode is not contraindicated for safety reasons, even with prolonged use [77].

### 10.5. Organs Effects

#### 10.5.1. Brain

Diagnostic ultrasonic investigation of the brain is limited because the bones of the skull block most of the transmission of US. However, transcranial Doppler (TCD) and transcranial color Doppler (TCCD) can be used to study the brain’s circulation and diagnose emboli, stenosis, vasospasm from subarachnoid hemorrhage, and other problems. Recording may be performed through the squama of the temporal bone, above the zygomatic arch, through the eyes, below the jaw, and from the post-auricular area. Brain ultrasound requires a low-frequency transducer (1–5 MHz) together with the transcranial Doppler software for image optimization [78]. US measurement of the optic nerve sheath diameter (ONSD) may aid in the diagnosis of intracranial hypertension (IH). A high-frequency (7–10 MHz) linear transducer is required [79]. US is useful in assessing the structure, function, and stability of the spine, also providing guidance in therapeutic interventions. US imaging of the spine typically requires the use of low-frequency US (2–5 MHz) and curved array transducers [80]. A high-frequency linear array probe (8–15 MHz) has to be used to imagine peripheral nerves [81].

Brain tissue has a relatively low absorption coefficient. However, as the skull temperature increases during ultrasound exposure, the temperature of the adjacent brain increases through conduction mechanisms [82]. This phenomenon is particularly important in the fetus when using the Doppler US modality.

In addition to these indirect thermal effects, US also causes direct neural effects. High-intensity focused US can produce destructive lesions in the brain [83]. Focused US beams that create therapeutic lesions in the subthalamic nucleus have been proposed to treat motor features of Parkinson’s disease [84]. The same technique has also been proposed for the treatment of some brain tumors [85].

Histological analysis on mammalian peripheral nerves revealed that US exposure might lead to neuronal and myelin destruction in the spinal cord [86]. Myelin, and especially the smaller myelinated fibers, are the structures most sensitive to US injury, thus leading to impaired neural conduction [87]. The damage seems to depend on both the thermal effect and the cavitation phenomenon [88,89]. Sodium and potassium channels open with increases in temperature during exposure to US, thus affecting conduction velocity [90]. Auditory evoked potentials can also be transiently suppressed after exposure to US in the diagnostic interval [91].

#### 10.5.2. Eye

Both B-mode and Doppler sonography have been used effectively and safely to diagnose many ophthalmic conditions, including intraocular or periorbital foreign bodies, globe rupture, lens dislocation, retinal and vitreous detachment and hemorrhage, neoplasm, and vascular pathologies [92,93]. However, exposure levels exceeding those encountered in diagnostic systems are capable of damaging the ocular structures.

The cornea and the lens of the eye, due to the lack of blood vessels and the large amount of collagen in their structures, are more prone to the absorption of US energy and, thus, are more likely to develop increased temperature during prolonged US exposure. Lizzi et al. [94] demonstrated that permanent lesions of the retina, choroid, and sclera could be produced with the use of focused US at 9.8 MHz in a rabbit model. Transient chemosis, conjunctival injection, and occasional hemorrhage were also reported. The different degrees of lesions depended on the duration and intensity of exposure. This evidence, in addition to histological examination of tissue samples, suggested that thermal mechanisms are the principal cause of permanent tissue alterations. High-intensity US is clinically used to fragment and emulsify the lens during cataract surgery. Corneal endothelial damage is a known risk of ultrasound phacoemulsification [95]. Both thermal and cavitation-related mechanisms are believed to be responsible for corneal endothelial damage induced by high-intensity focused US [96].

Due to the concern about intraocular damage, the FDA limited ocular exposure to a spatial peak, average temporal intensity (I_SPTA_) of 50 mW/cm^2^ [19]. Similarly, the BMUS recommended limiting thermal and mechanical indices to less than 1 and 0.7, respectively, during ocular exposure to ultrasound [29].

#### 10.5.3. Heart

Echocardiography is routinely used in the diagnosis, management, and follow-up of patients with any suspected or known heart diseases. It provides helpful information on the size and shape of the heart, wall motion, ejection fraction, diastolic function, and assessment of valves [97].

US has been shown to act on both cardiac rhythm and contractility. Single high-amplitude US pulses used in lithotripsy can produce premature ventricular contractions in frogs [98]. The most sensitive phase of the cardiac cycle to produce a premature contraction with an acoustic pulse is during the diastole. Therefore, in clinical practice, the administration of US pulses is synchronized with the electrocardiogram (ECG) to avoid effects on heart rhythm. In frogs and mice, the “threshold” for producing a premature contraction with a single 5 ms US pulse at 1.2 MHz was about 2–5 MPa. This threshold showed increases with decreasing pulse duration and increasing frequency [99]. However, the relatively long pulse durations and high-pressure amplitudes required to produce this effect are not characteristic of exposures used for diagnostic US.

Gaseous microbubbles or acoustic cavitation may play a role in the generation of premature cardiac contractions with US [100,101]. Premature ventricular contractions have occurred in men exposed to diagnostic US when an experimental contrast agent was present in the blood [102]. In laboratory animals injected with contrast agents, the threshold for producing a premature contraction with a single 10 μs pulse of US at 1 MHz was of the order of 1 MPa [103]. Microvascular damage has also been observed in hearts exposed to US contrast medium. However, the relationship between arrhythmia generation and microvascular effects is uncertain [34,103].

A single high-amplitude US pulse can also affect cardiac contractility. In frogs, pulsed US has been shown to produce a decrease in the maximum aortic pressure, an abnormal relaxation, or a combination of both effects. The threshold for this effect was about 5 MPa using a 5 ms pulse at a frequency of 1.2 MHz [99]. Aortic pressure returned to control values within one or two beats after US exposure, suggesting that the contractile components of the tissue were not damaged. For this effect, an US pulse is most effective when the heart is scanned during systole. A number of experimental investigations suggest that ARF is the main acoustic mechanism acting on cardiac contractility [104].

#### 10.5.4. Skeletal Muscle

Diagnostic US is useful for the evaluation of normal and pathological muscle tissue. It is used to help diagnose sprains, strains, and tears [105]. Atrophy can be objectified by measuring muscle thickness, while degenerative changes, such as infiltration of fat and fibrous tissue, increases muscle echo intensity [106].

US-induced temperature elevation could damage muscular structures. In vitro experiments on chick embryonic skeletal muscle cells have shown that low-intensity pulsed ultrasound wave (pulsed 0.5 W/cm^2^ intensity for 5 min) induced cell proliferation, low-intensity continuous ultrasound wave (continuous 0.5 W/cm^2^ intensity for 5 min) induced muscle differentiation, and high-intensity pulsed ultrasound wave (pulsed 1.0 W/cm^2^ intensity for 10 min) induced cellular death [107]. Miller and Quddus [108] observed the induction of petechiae in the abdominal muscles of mice using diagnostic ultrasound associated with the use of contrast microbubbles. The petechiae number was approximately proportional to the contrast agent dose. Skyba et al. [109] used intravital microscopy to visualize bioeffects resulting from contrast gas body destruction using diagnostic ultrasound in the spinotrapezious muscle in rats. Harmonic mode imaging of muscle was performed with a phased array diagnostic ultrasound system and a mean frequency of 2.3 MHz. A single-image frame was obtained with MIs of 0.4, 0.5, 0.7, and 1.0 for each animal. After exposure, the muscle was examined for microvessel rupture and dead cells (i.e., stained with Propidium Iodide). Capillary rupture sites and stained cells were absent at an MI of about 0.4 but increased rapidly for higher MI values.

In recent decades, low-intensity pulsed ultrasound (LIPUS) has been suggested to have a role in assisting muscle restoration after injuries. LIPUS is a form of ultrasound therapy typically used in rehabilitation medicine that employs low-intensity radiation in pulsed-wave form. Spatial-average temporal-average intensities (ISATA) used in LIPUS are determined by the amplitude of the “on” period, varying from 0.02 to 1 W/cm^2^ at frequencies ranging from 1 to 3 MHz. Low-intensity exposure minimizes the possibility of thermal effects. Shu et al. [110] demonstrated the effectiveness of LIPUS treatment at different doses (0.25 W/cm^2^, 0.5 W/cm^2^, and 0.75 W/cm^2^) in a rat contusion injury model. Piedade et al. [111] found that US was able to stimulate myoregeneration and collagen deposition in an experimental rat model of lacerative gastrocnemius muscle lesion. Moreover, Karnes et al. [112] reported that LIPUS treatment increased fibroblast proliferation, capillarization, and myofiber formation in a rat muscle injury model. Even if the real mechanism underlying LIPUS effectiveness is not yet understood, it is plausible to hypothesize a role for non-thermal phenomena, such as acoustic streaming, microstreaming, and mechanical stimulation. The vibratory effect of ultrasonic energy on the cell surface can activate some mechanically sensitive ion channels and induce changes in cell membrane permeability, thus stimulating the transport of second messenger substances, such as calcium, across the cell membrane. Second messengers can then up-regulate the production of growth factors and other signaling molecules, promoting the recovery of injured muscles. Reher et al. [113] observed that LIPU could stimulate the production of interleukin 8 (IL-8), basic fibroblast growth factor (bFGF), and vascular endothelial growth factor (VEGF). VEGF can promote muscle fiber angiogenesis. bFGF can induce activation of quiescent satellite cells (located between the basal lamina and cell membrane of skeletal muscle fibers) into myoblasts, thereby contributing to muscle damage repair. Montalti et al. [114] showed that LIPUS exposure increased Cyclooxygenase-2 (COX-2) expression in the muscles of rats, and this finding was associated with the formation of new muscle fibers and more organized tissue structure in treated animals. A possible explanation relies on the fact that COX-2 is a key enzyme in converting arachidonic acid into prostanoids or prostaglandins. In particular, prostaglandin E2 (PGE2) is essential for efficacious skeletal muscle stem cell function, augmenting regeneration, and strength. Sugitta et al. [115] also observed that the stimulation of the abductor muscle in a rabbit model with increasing LIPUS intensity (between 0.21 and 0.48 W/cm^2^) resulted in increasing NO levels through the induced nitric oxide synthase (iNOS)-dependent pathway, leading to increased vasodilation and blood flow independently of thermic effects. However, the effectiveness of LIPUS as a modality that can modulate muscle regeneration after muscle injury is still debated because some investigations have demonstrated no positive effect [116,117,118].

#### 10.5.5. Bone

Diagnostic US performed on post-trauma soft tissues has shown to be able to identify occult fractures undetected by the previous X-ray [119,120,121]. Due to the different acoustic impedance between soft tissues and the bone cortex, US only allows the evaluation of the bone surface, which appears as a hyperechoic continuous line. A localized interruption of this hyperechoic line in an US is a hallmark of acute fracture. Associated findings are abnormalities of the periosteum (subperiosteal hematoma), adjacent soft tissues (local hematoma and edema), and joints (articular effusion). US plays a complementary role in the assessment of osteomyelitis, detecting subperiosteal effusion, as well as abscesses and fistula in the adjacent local soft tissues. Osteochondromas can be visualized as localized outgrowths of bone, in continuity with the normal cortex and medullary, covered by a cartilaginous cap. On the contrary, US has very limited capabilities in assessing other bone tumors [122].

The Food and Drug Administration (FDA) approved the use of low-intensity pulsed ultrasound (LIPUS) to accelerate the healing of fresh fractures in 1994 and for the treatment of non-unions in 2000 [123]. LIPUS has been shown to significantly increase the rate of fracture repair in various animal models [124,125,126]. Furthermore, several clinical trials have confirmed the efficacy of ultrasound in accelerating fracture healing in humans [127,128]. Effective exposure in accelerating fracture healing consists of a 200 µs pulsed burst of 1.5 MHz US waves repeating at 1 kHz and delivering 30 mW/cm^2^ spatial-average and temporal-average (SATA) applied at the fracture site for 20 min per day [123]. Mechano-transduction pathways seem to be involved in cell responses. These include the activation of MAPK and other kinases pathways, gap-junctional cell-to-cell intercellular communication, up-regulation and clustering of integrins, the involvement of the COX-2-PEG2 and the iNOS/NO pathways, and activation of angiotensin II type I (ATI) mechanoreceptor [124]. Although the mechanisms by which LIPUS can trigger these effects remain unknown, the low-intensity exposures of LIPUS treatment make thermal effects unlikely. Possible mechanisms may, therefore, include mechanical effects, such as acoustic radiation force, acoustic streaming, and propagation of surface waves [124].

Dalecki et al. [129] reported that lithotripter pulses with amplitudes less than 1 MPa could produce vascular damage in late-term murine fetuses exposed in utero. Hemorrhages occurred only in tissues near developing bone (head, limbs, and ribs), whereas soft tissues distant from bone were unaltered. Moreover, no damage was observed in murine fetuses exposed to lithotripter pulses at a stage of gestation prior to bone formation. Successively, the same group of authors demonstrated that hemorrhage to tissues near developing fetal bone could also result from exposure of murine fetuses to pulsed ultrasound [130]. Hemorrhage occurred most frequently in the fetal head. The pressure threshold for producing hemorrhage to the fetal head was about 2.5 MPa negative pressure, and the threshold increased with increasing frequency (2.4 and 3.6 MHz). This threshold is above the current output limits of diagnostic devices. The physical mechanism for damage in tissues near fetal bone as a result of low-amplitude lithotripter fields or pulsed US has yet to be understood. Thermal effects are not justified by US parameters employed in both studies. The authors hypothesized a purely mechanical effect resulting in damage of fragile fetal vessels between partially ossified bones and surrounding tissues.

#### 10.5.6. Lung

A healthy lung is scarcely penetrable for diagnostic US due to its air content. Because of the high difference in acoustic impedance, more than 96% of the US beam is reflected at the interface between chest wall tissues and pulmonary air interface, resulting in the so-called “hyperechoic pleural line”. However, US in the lung may aid in the detection of pleural effusions, pleural alterations, and peripheral pulmonary consolidations adhering to the superficial pleura. Furthermore, US has been demonstrated as safe and effective in guiding thoracentesis and percutaneous needle biopsy of pleural lesions and US-accessible peripheral lung consolidations. Lung examination is generally performed with a low multi-frequency convex probe (3.5–5 MHz). A high-frequency linear probe (8–12.5 MHz) may be used in order to obtain more detailed information on the appearance of the hyperechoic pleural line [131].

Child et al. [132] discovered the induction of pulmonary capillary hemorrhage using pulsed US more than 20 years ago. This bioeffect has been extensively studied in mice, rats, rabbits, and pigs. In a major review (AIUM, 2000 [37]), threshold trends for its occurrence were found to be near an MI value of 0.63. However, only one study each has been conducted on monkeys and humans. Tarantal and Canfield [133] showed some evidence of pulmonary hemorrhage in monkeys utilizing a commercial diagnostic US machine with a linear array. The hemorrhage itself mostly originated from the microvasculature of the visceral pleura. However, US-induced lung hemorrhage resulting from alveolar injury and congestion in alveolar capillaries was also documented. In contrast with experimental animals, humans did not appear to develop lung hemorrhage as a result of US exposure. A study of 50 humans was conducted by examining the left lungs after routine intraoperative transesophageal echocardiography with the highest MI output of 1.3. The surgery was mostly for coronary artery bypass grafting. None of the patients developed lung hemorrhage [134]. This result was considered to be uncertain with regard to pulmonary hemorrhage thresholds because the actual direct exposure of the lung to the highest pressure amplitudes was uncertain for these examinations.

In 2000, O’Brien et al. [135] observed that the occurrence of pulmonary hemorrhage was not inversely correlated with increased overpressure (i.e., hydrostatic pressure), which was used to reduce or eliminate the negative total pressure during the pulse. Furthermore, in another study, O’Brien et al. [136] observed that the injection of microbubbles did not increase the occurrence of pulmonary capillary hemorrhage during US exposure in rats. Similarly, in the study by Raeman et al. [137], the injection of contrast-agent microbubbles in mice did not increase the sensitivity to pulmonary hemorrhage compared to saline injection. These authors concluded that the mechanism of US-induced lung hemorrhage might not be directly related to inertial cavitation.

However, the presence of air in the lungs seems to be crucial for lung hemorrhage. In 2002, O’Brien et al. [138] observed that while pulmonary hemorrhage occurred in pregnant mice for 3.1 MHz pulsed US at 2 MPa, no signs of damage were found in the fetal lung, which lacks gas, even at 20 MPa. Transmission into the lungs varies with the degree of inflation, with deflated lungs more easily damaged than half-inflated lungs. The pulmonary hemorrhage effect did not appear to be caused by the heating of the lung tissue, which might be expected from the relatively high absorption coefficient of lung tissue [139]. In this regard, Hartman et al. [140] showed that the temperature elevation produced at the lung’s surface in mice was approximately only 1 °C after 5 min for a 4.2 MHz continuous wave of 1 W/cm^2^. On the other hand, Bayley et al. [141] showed that peak compressional pressure amplitudes ranging from 1 to 5 MPa are capable of producing hemorrhage in murine lungs in a strongly pressure-dependent manner. This study emphasizes that the threshold for lung hemorrhage is lower than other non-gas-containing tissues and that currently available diagnostic US devices may theoretically produce such injury.

The specific problem of pulmonary bioeffects from diagnostic US remains, therefore, difficult to address in terms of patient safety. However, morphologic studies have suggested that shorter, thinner, and less distensible terminal airways, a reduced alveolar surface tension and capillary surface area, and a lower thickness of the visceral pleura may determine a greater predisposition of mice, rats, and rabbits to the development of ultrasound-induced lung hemorrhage compared to primates and humans [11].

#### 10.5.7. Digestive Tract

US is a noninvasive imaging test that is currently used in the complementary diagnosis of a wide range of abdominal issues safely.

The digestive tract naturally contains gas. The main segments of the digestive tract that may be exposed to diagnostic US during abdominal examination include the stomach, small intestine, large intestine, cecum, and colon. All these organs share a similar laminar structure organized in four concentric tissue layers. From the outside to the inside, these are the serosa (i.e., the peritoneum), the muscularis propria (consisting of an outer longitudinal and an inner circular smooth muscle layer), the submucosa, and the mucosa (organized in muscular mucosae, lamina propria, and epithelium). The inner mucosal epithelium is supported by a highly vascularised capillary bed within the lamina propria and looks into the internal lumen. Gaseous bodies of different sizes are present within the lumen in each digestive segment. Their amount and dimension can vary from segment to segment due to peristalsis.

Pulsed US exposure can result in the occurrence of intestinal hemorrhage in mice. In a study from Dalecki et al. [142], mice were irradiated using focused sources operating from 0.7 to 3.6 MHz for 5 min. The pressure threshold varied from 1 to 4 MPa in the frequency range. These thresholds are close to the current upper limit of diagnostic imaging devices. Higher frequencies were less effective in producing intestinal hemorrhage than lower frequencies. A temperature increment of only 1–2 °C degrees was documented at the highest exposure levels. These results suggested that the production of intestinal hemorrhage is consistent with a cavitation-related mechanism of action of pulsed US. Furthermore, lesions appeared to be associated with the mucosal surface and not the serosal surface of the intestine. Hemorrhage occurred in the mucosal-submucosal layer, and blood was evident within the lumen. Hemorrhage in the murine intestine has also been reported to result from exposure of mice to lithotripter fields where the mechanism is clearly non-thermal [143,144]. The presence of gas in the intestine seems to be necessary to produce intestinal hemorrhages with ultrasound. In 1995, Dalecki et al. [145] exposed pregnant mice to a lithotripter field of higher-pressure amplitude than the threshold causing intestinal damage in adult mice. Although the gas-containing maternal intestines were extensively damaged, the gas-free intestines of fetal mice were not.

Lehmann and Herrick [146] reported the production of petechial hemorrhage in the abdomen of mice exposed to continuous US waves at 1 MHz. The mechanism of damage was attributed to cavitation by these authors because the lesions had an appearance similar to that of mechanical injuries on histological examination. On the contrary, in the study by Miller and Thomas [147], the induction of petechiae by continuous US in mice intestines was associated with the occurrence of hyperthermia. These authors, therefore, concluded that the US-induced petechiae were attributable to heating and not cavitation. In addition, these authors observed that the petechiae arising from heating with continuous US were quite different from the intestinal hemorrhages induced by lithotripter shock waves. Ultrasonically-induced thermal petechiae were characterized by leakage of blood from the capillary vessels into the lamina propria (with no evident tissue destruction), while the shock-wave-induced hemorrhages involved blood flowing into the lumen of the intestine with histologically obvious tissue destruction and clotting.

The presence of US microbubble contrast agents in the vascular system has been shown to increase the extent of damage to the murine intestine exposed to both continuous and pulsed US. In general, the threshold for effect increases with increasing frequency and decreasing pulse duration [148,149]. Harmful side effects have not been reported in humans associated with the clinical application of continuous wave US (typical of therapeutic applications) or pulsed US (typical of diagnostic applications). Currently, there are no data indicating significant differences in the gut between or within species that could potentially influence susceptibility to ultrasound-induced intestinal haemorrhage [11]. These differences must be investigated.

## 11. Conclusions

To date, there are no reports in the literature of actual biological damage in patients undergoing diagnostic US. This has led to considering US to be a safe and well-tolerated diagnostic method with low biological risk. At present, there are not enough experimental data to concretely define the risk from exposure to low-power diagnostic US. The EFSUMB [57] and AIUM [58] have officially declared that acoustic power values commonly used in US diagnostics are safe. However, the use of TI and MI as “safety indices” has clear limitations based on their derivation from calculations giving estimates of “worst-case” average values in tissue model assumptions to the human being. Furthermore, the possibility of non-linear effects is not considered, and TI and MI indices may be inappropriate for new imaging techniques. For example, the use of color flow or pulsed Doppler US may increase the potential temperature rise by an “unknown factor”, and contrast-enhanced US (CEUS) lowers the cavitation threshold. ARFI SWE mode requires the employment of a MI no lower than 1.0 (from 1.3 to 1.6) and much longer pulses than those used in standard ultrasound (30–300 ms) to cause tissue displacement. In addition, the effects of very short-duration focused ARF energy peaks remain unknown and should be explored by further experimental studies. Despite no harmful biological effects yet demonstrated in humans with the new imaging modalities, in-depth knowledge of the potential risks of ultrasound-mediated biological effects is required. While the biological effects of diagnostic ultrasound continue to be investigated in research laboratories, physicians should reduce ultrasound exposure to as little as possible to obtain useful diagnostic information.

## Figures and Tables

**Table 1:** Applications of ultrasound across different medical disciplines.

Medical Discipline	Application
Abdominal	To diagnose the cause of abdominal pain or discomfort; To evaluate liver, gallbladder, bile ducts, pancreas, spleen, and abdominal aorta; To identify occlusions, bleeding, and suspicious lesions in the abdominal organs.
Anaesthesiology	To guide the delivery of local anesthetics to the target area while avoiding structures that might be damaged by the needle; To guide peripheral and central venous catheterization.
Breast	To help the identification and study of breast lumps; To guide biopsy of suspicious breast lesions.
Cardiology	To detect pericardial effusion, cardiac wall motion abnormalities, and vascular stenoses; To estimate ejection fraction (EF) and pulmonary artery pressure (PAP).
Dentistry	To identify carious lesions, tooth fractures or cracks, periodontal bony defects, and maxillofacial fractures; To remove tartar and clean teeth.
Endocrinology	To evaluate the thyroid and parathyroid glands; To guide biopsy of suspicious thyroid lesions.
Gynecology	To study the female pelvic organs, mainly the ovaries, fallopian tubes, uterus, bladder, adnexa, and recto-uterine pouch; To help identify malignant and endometriosis lesions.
Musculoskeletal	To study the morphology of joints and muscle fibers; To increase blood flow, reduce pain and swelling, and promote healing in muscular sprains and strains and in the treatment of common issues such as tennis elbow, ankle sprain, tendonitis, rotator cuff, shoulder pain, and many others (Shockwave Therapy).
Nephrology	To evaluate the kidney and detect the presence, number, and size of kidney stones; To break large kidney stones into smaller fragments that can easily pass through the urinary tract (lithotripsy).
Obstetrics and Pregnancy	To provide a high-quality two-dimensional image of the embryo or the fetus in real-time; To detect any abnormalities in the growth of the fetus or any other problems associated with the pregnancy.
Ophthalmology	To diagnose ophthalmic diseases, including intraocular or periorbital foreign bodies, globe rupture, lens dislocation, retinal and vitreous detachment and hemorrhage, neoplasm and vascular pathologies; To fragment and emulsify the lens during cataract surgery (phacoemulsification).
Pulmonology	To identify pleural effusion, lung consolidations, and morphological and movement alterations of the hyperechoic pleural line; To guide thoracentesis and percutaneous biopsy of lung lesions.
Urology	To examine the urinary tract for any defects and to identify bladder tumors; To break large urinary tract stones into smaller fragments.

**Table 2:** Different types of ultrasound probes with their characteristics and uses.

Type	Characteristics and Uses
**Linear**	Active elements are arranged in a line; They generate a rectangular scan field; They work at high frequencies (from 7.5 to 16 MHz); Used to study superficial structures (i.e., muscles, tendons, breast, thyroid).
**Convex**	Active elements of the transducer are arranged on an arc of circumference; They generate a trapezoidal-shaped scan area; They work at medium and low frequencies (from 3.5 to 5 MHz); Used for the study of deeper structures (i.e., visceral organs).
**Sectorial**	Active elements are aligned in a short band or rectangle configuration; They generate a fan-shaped beam; They work at low frequencies (from 2 to 3.5 MHz); Usually used for cardiac examination.
**Endocavitary**	They can be linear or convex; Used for intrarectal or intravaginal examinations; They work at medium frequencies (from 5 to 7.5 MHz).

**Table 3:** Density, acoustic impedance, and longitudinal wave velocity of US in biological tissues.

Tissue	Density (Kg/m^3^)	Longitudinal Wave Velocity (m/s)	Acoustic Impedance (Kg/m^2^s) × 10^6^
Air	1.2	330	0.0004
Lung	400	440–500	0.18–0.20
Adipose tissue	920	1460	1.35
Water	1000	1480	1.48
Liver	1060	1550	1.64
Spleen	1060	1560	1.65
Blood	1060	1560	1.62
Kidney	1040	1560	1.62
Muscles	1070	1590	1.70
Connective tissue	1120	1610	1.80
Cartilage	1100	1665	1.85
Skin	1150	1730	1.99
Bone	1380–1810	2700–4100	3.75–7.38

**Table 4:** Attenuation coefficients of various media or tissues.

Medium/Tissue	Attenuation Coefficient: dB/cm at 1 MHz
Water	0.0022
Blood	0.15
Skin	0.35
Spleen	0.40
Liver	0.50
Adipose Tissue	0.63
Heart	0.52
Breast	0.75
Skeletal muscles: Perpendicular fibers Parallel fibers	0.96 1.40
Kidney	1.00
Connective Tissue	1.57
Air	7.50
Bone	15.00
Lung	40.00

## Data Availability

Not applicable.
